# A novel *Phytopythium* species causing root rot of *Salvia miltiorrhiza* in China: pathogenicity, molecular identification, and biocontrol potential

**DOI:** 10.3389/fpls.2026.1803698

**Published:** 2026-04-24

**Authors:** Haotong Li, Xiaojian Chen, Langjun Cui, Yi Qiang, Bixin Bai

**Affiliations:** National Engineering Laboratory for Endangered Medicinal Resource Development in Northwest China, Key Laboratory of Medicinal Resources and Natural Pharmaceutical Chemistry of Ministry of Education, College of Life Sciences, Shaanxi Normal University, Xi’an, China

**Keywords:** biological control, ITS, Phytopythium shangluoense, root rot, Salvia miltiorrhiza, Streptomyces fungicidicus

## Abstract

In the Shangluo region of China, *Salvia miltiorrhiza* plants have been observed with black-brown necrosis on the aerial parts, while the roots retain an intact epidermis but display internal rot that can be easily crushed by hand. A pathogen was consistently isolated from the diseased roots and, based on colony morphology, was identified as a member of the genus *Phytopythium*. Molecular identification based on partial sequences of four gene regions—the nuclear ribosomal internal transcribed spacer (ITS), mitochondrial cytochrome c oxidase subunits I and II (*cox* I and *cox* II), and *β-tubulin*—confirmed the morphological classification and revealed that the isolate represents a previously undescribed *Phytopythium* species, designated *Phytopythium shangluoense* after its geographic origin. Detached root inoculation assays demonstrated that *P*. *shangluoense* could cause complete root rot of *S*. *miltiorrhiza* within 15 days. Within the temperature range of 13–28 °C, seedling disease incidence reached 100% within three days after inoculation. Additionally, a set of genus-specific primers based on the ITS region was designed and validated, allowing sensitive and accurate discrimination of *Phytopythium* spp. from other known root pathogens of *S*. *miltiorrhiza*. Furthermore, dual culture assays and subsequent pot assays demonstrated that *Streptomyces fungicidicus* strain FYA1 exhibits significant inhibitory effects against *P*. *shangluoense*, achieving an efficacy of 80% against root rot caused by this pathogen. This study provides the first confirmed evidence of the isolation and designation of *P. shangluoense* and demonstrates that it is an aggressive oomycete plant pathogen capable of independently causing root rot and plant death in *S*. *miltiorrhiza*, offering critical insights for early diagnosis and integrated disease management.

## 1 Introduction

*Salvia miltiorrhiza* Bunge, a perennial herbaceous plant belonging to the genus *Salvia* in the family Lamiaceae, is widely used in the treatment of cardiovascular diseases due to the abundance of active compounds such as tanshinones and salvianolic acids in its dried roots and rhizomes (Guo et al., 2014; Huang et al., 2024). In recent years, the rapid increase in market demand has led to a sharp expansion in the cultivation area of *S*. *miltiorrhiza* (Lu, 2021). To meet the increasing demand, *S*. *miltiorrhiza* has often been cultivated under intensive and continuous cropping systems. However, the long-term continuous cropping has led to increasingly severe soil-borne diseases, among which root rot poses the most significant threat (Jiang et al., 2024).

Root rot caused by species of the oomycete genus *Phytopythium* represents an emerging group of soil-borne diseases that pose a serious threat to a wide range of economically important crops, forest trees, and ornamental plants worldwide. This genus was formerly classified within *Pythium* but was subsequently redefined as a distinct oomycete genus based on morphological characteristics and phylogenetic analyses (de Cock et al., 2015). *Phytopythium vexans* is the most representative and widely distributed pathogenic species within the genus and has been confirmed to infect numerous economically important hosts. For example, *P. vexans* has been identified as the causal agent of root rot in *Cinnamomum camphora* (Xiao et al., 2023), *Ginkgo biloba* (Panth et al., 2021), *Rhododendron simsii* (Liu et al., 2025), apple and pear (Jabiri et al., 2021), and kiwifruit (Türkkan et al., 2022). In addition, *Phytopythium helicoides* has been reported as an important pathogen causing root rot in begonia (Yang et al., 2013) and chrysanthemum (Lee and Choi, 2025). These studies highlight the broad host range and increasing impact of *Phytopythium* species on medicinal plants and horticultural crops.

Root rot caused by *Phytopythium* species typically manifests as water-soaked brown necrotic lesions on the root surface, cortical decay, browning of internal vascular tissues, and eventual complete root rot (Panth et al., 2021; Parajuli et al., 2024). In regions where the disease is prevalent, substantial economic losses have been reported. For instance, in kiwifruit-producing areas of Türkiye, *P. vexans* was isolated at a frequency of up to 34.3% and, together with other oomycete species, contributed to plant decline and mortality (Türkkan et al., 2022). In apple and pear orchards in Morocco, disease incidence associated with this pathogen reached 100% in certain areas (Jabiri et al., 2021). Collectively, these findings underscore the increasing threat posed by *Phytopythium* species to medicinal and economically important crops, with considerable economic consequences.

The reported pathogens causing root rot in *S*. *miltiorrhiza* primarily include multiple species of the genus *Fusarium*, such as *Fusarium solani*, *F*. *equiseti*, *F*. *oxysporum*, and *F*. *proliferatum* (Pu et al., 2022; Song et al., 2023; Yang et al., 2021a, 2021b). These pathogens exhibit strong infectivity under high-temperature and high-humidity conditions (15–25 °C), and are capable of killing seedlings within three days of inoculation (Doohan et al., 2003; Liu et al., 2023). However, investigations of oomycete pathogens, particularly species of the genus *Phytopythium*, associated with *S*. *miltiorrhiza* root rot remain limited.

Shangluo, located on the southern slopes of the Qinling Mountains in China, is one of the traditional geo-authentic production areas of *S*. *miltiorrhiza* (Brinckmann, 2013; Luo et al., 2021). In recent years, a root rot symptom distinct from previously reported cases has been observed in this region: the aboveground parts exhibit dark brown necrosis and dieback, while the root epidermis remains intact and the internal tissues become soft and decayed, suggesting the possible presence of a novel pathogen. Nevertheless, the putative pathogen has not yet been isolated or reported. We hypothesized that this distinct root rot is caused by an unreported oomycete pathogen, which can be differentiated from known species by significant genetic divergence in multi-locus phylogeny (>3% sequence divergence in ITS region and >5% in *cox* genes) and by fulfilling Koch’s postulates (Robideau et al., 2011; Schoch et al., 2012). We further hypothesized that a PCR-based detection method targeting species-specific regions of the ITS region will achieve ≥95% diagnostic accuracy in discriminating this novel pathogen from other root pathogens of *S. miltiorrhiza* (Cooke et al., 2000; Hyde et al., 2017). To test these hypotheses, we collected symptomatic *S*. *miltiorrhiza* plants from the Shangluo district, isolated the putative pathogens, and assessed their pathogenicity by fulfilling Koch’s postulates. The taxonomic position was determined based on morphological characteristics and multigene phylogenetic analyses, leading to the identification of a novel *Phytopythium* species. A genus-specific detection method targeting the ITS region was developed, and the biocontrol potential of strains from our laboratory collection was evaluated against the newly identified pathogen. This study not only expands the known pathogen spectrum associated with *S*. *miltiorrhiza* root rot but also provides a theoretical foundation for early diagnosis and environmentally friendly disease management strategies.

## 2 Materials and methods

### 2.1 Isolation and purification of the pathogen

Roots of *S*. *miltiorrhiza* plants exhibiting typical root rot symptoms were collected from cultivation fields in Baoji, Shangluo, and Xianyang, Shaanxi Province. Pathogenic organisms were isolated from representative root rot tissues by isolation from the lesion margin (Agrios, 2005; Erwin and Ribeiro, 1996). Small tissue sections (approximately 0.5×0.5 cm) were excised from the junction between lesioned and healthy tissue. The sections were first rinsed under running tap water for 10–30 min, followed by a brief rinse with sterile distilled water (SDW). Surface sterilization was then performed by sequential immersion in 70% ethanol for 1 min and 20% sodium hypochlorite solution for 10 min. Afterward, the tissues were rinsed five times with SDW to remove residual disinfectants. Finally, the tissue pieces were blotted dry on sterile filter paper and placed onto potato dextrose agar (PDA) plates (Diabankana et al., 2024; Leslie and Summerell, 2008). The plates were incubated at 28 °C in darkness for 24–48 h. For purification, individual hyphal tips were isolated and transferred onto fresh PDA plates. This subculturing procedure was repeated three to five times until axenic strains was obtained (Howard and Aist, 1979).

### 2.2 Morphological identification

To identify the isolate SLDS1, purified strains were first cultured on PDA and V8 agar plates at 28 °C. Colony growth was monitored by measuring the colony diameter daily for 4 days, and macro-morphological features were documented. Subsequently, for microscopic characterization, isolate SLDS1 was grown using the slide culture method for 48 h. Hyphal morphology and spore characteristics were then examined under a light microscope. A preliminary identification was made by comparing both the colonial and microscopic morphological characteristics with descriptions in authoritative mycological references, including *Plant Pathogenic Fungology* (Barnett and Hunter, 1972).

### 2.3 Molecular biological identification

Approximately 200 mg of fresh mycelia scraped from 4- to 5-day-old cultures was used for genomic DNA extraction following the CTAB method (Gill et al., 2025). PCR amplifications were performed using this DNA as the template (Cenis, 1992). For preliminary identification, the internal transcribed spacer (ITS) region of ribosomal DNA was amplified using the universal fungal primers ITS4 and ITS5 (Grassi et al., 2018). For further species-level identification within the genus *Phytopythium*, specific primer sets targeting the *β-tubulin*, cytochrome c oxidase subunit I (*cox* I), and subunit II (*cox* II) gene fragments were employed (**Supplementary Table 1**) (Chen et al., 2016; Villa et al., 2006). PCR reactions were carried out in a total volume of 25 µL, containing 12.5 µL of PrimeSTAR Max Ver.2 Premix (2×) (Takara, Beijing, China; Cat. No. R047A), 1 µL of each forward and reverse primer (10 µM), 2 µL of DNA template, and SDW to volume. The thermal cycling profile consisted of an initial denaturation at 98 °C for 3 min; followed by 35 cycles of denaturation at 98 °C for 15 s, annealing at 52 °C for 15 s, and extension at 72 °C for 30 s; with a final extension at 72 °C for 5 min. The PCR products were verified by electrophoresis on 1.5% agarose gels and subsequently sent for bidirectional sequencing. The obtained sequences were subjected to BLASTn (https://blast.ncbi.nlm.nih.gov/Blast.cgi) homology searches against the NCBI GenBank database (Raja et al., 2017). A phylogenetic tree was constructed using the Maximum Likelihood (ML) method in MEGA 11 software based on the concatenated dataset of the gene fragments. The robustness of the tree was assessed by bootstrap analysis with 1,000 replicates (Kumar et al., 2016; Naruya and Masatoshi, 1987; Tamura et al., 2021; White et al., 1990).

### 2.4 Pathogenicity assay

#### 2.4.1 Inoculation of seedlings

Healthy seeds of *S. miltiorrhiza* were surface-disinfected by immersion in 75% ethanol for 1 min, rinsed once with SDW, treated with 10% hydrogen peroxide (H_2_O_2_) for 10 min, and finally rinsed three times with SDW. The disinfected seeds were soaked in SDW at room temperature for 8 h to promote germination. They were then placed on moistened sterile filter paper and incubated in a growth chamber at 25 °C under a 16 h light/8 h dark photoperiod for 12 days until seedlings developed two to four true leaves. Uniform seedlings were arranged on freshly prepared V8 agar plates. For inoculation, mycelial plugs (4 mm in diameter) taken from the margins of 3-day-old fungal colonies using a sterile cork borer, were placed in contact with the seedling roots (Lu et al., 2023). Seedlings inoculated with sterile PDA plugs served as the negative control. The inoculated seedlings were incubated at 13-25 °C (simulating diurnal temperature fluctuation) for 6 days and monitored daily for symptom development.

#### 2.4.2 Inoculation of detached root segments

The sterile seedlings were transplanted into pots containing an autoclaved mixture of vermiculite and potting soil (1:1, v/v) and cultivated under the same conditions for six months until the taproot diameter reached approximately 0.6 cm. Healthy roots were harvested, rinsed under running tap water to remove adhering substrate, and surface-disinfected (70% ethanol for 30 s, followed by three SDW rinses). The roots were cut into segments (0.6 cm in diameter, 5 cm in length). A longitudinal wound (approximately 1 cm long) was made in the middle of each segment using a sterile scalpel. A mycelial plug (4 mm in diameter, taken from the margin of a 3-day-old culture) was placed with the mycelial side facing down onto the wound, and the inoculation site was wrapped with Parafilm to maintain humidity. Root segments inoculated with sterile PDA plugs served as the control (Liu et al., 2022b; Yang et al., 2021b). All inoculated root segments were incubated at 25 °C for 15 days, after which rot symptoms were assessed.

Each experiment was conducted in a completely randomized design with three independent biological replicates, each comprising at least five seedlings or root segments. To fulfill Koch’s postulates, symptomatic tissues from all treatments were sampled for pathogen re-isolation (Reich, 1884; Wilkos et al., 2022). The re-isolated strains were compared morphologically and/or molecularly with the original inoculum to confirm their identity.

### 2.5 Design and validation of specific primers

Based on the ITS sequence obtained from the novel pathogen, multiple sequence alignment was performed using ClustalW against ITS sequences from key species within the same genus and other common pathogens on *S. miltiorrhiza* (e.g., *Fusarium* spp., *Rhizoctonia* spp.) (Kumar et al., 2016; Thompson et al., 1994). From the alignment results, unique and conserved regions specific to the novel pathogen were selected. Genus-specific primer pairs were designed targeting these regions using Primer Premier 5.0 software with the following default parameters: primer length of 18–24 bp, melting temperature (Tm) of 50-55 °C, GC content of 40-60%, and an expected amplicon size of 150–300 bp. To verify the specificity of the designed primers, PCR amplifications were performed in a 25 μL reaction volume using the 2× Rapid Taq Master Mix (Vazyme Biotech, Nanjing, China; Cat. No. P222-03-AA). Each reaction contained 12.5 μL of 2× Master Mix, 0.5 μL each of forward and reverse primers (10 μM), 1 μL of genomic DNA template (10 ng/μL) from each species included in the aforementioned alignment, and 10.5 μL of ddH_2_O to make up the final volume. The thermal cycling conditions were as follows: initial denaturation at 95 °C for 3 min; 35 cycles of denaturation at 95 °C for 30 s, annealing at 50 °C for 30 s, and extension at 72 °C for 30 s; followed by a final extension at 72 °C for 5 min. The PCR products were analyzed by electrophoresis on 1.5% agarose gels. Specific amplification was expected to yield a single band of the correct size only in the lane containing DNA of the target novel pathogen. To validate the specificity of the designed primers (Ps-F/Ps-R), a test panel was assembled, including three oomycete species—*Phytophthora capsici*, *Phytophthora parasitica*, and *Phytophthora sojae*; three *Fusarium* genus—*Fusarium acuminatum*, *Fusarium solani*, and *Fusarium oxysporum*; as well as *Alternaria alternata* and *Sclerotinia sclerotiorum*. Genomic DNA from all these isolates was used as template for PCR amplification with the primer pair Ps-F/Ps-R under the conditions described above. Specificity was confirmed by the absence of amplification products in all non-target species. The GenBank accession numbers for the sequences used in this specificity validation are provided in **Supplementary Table 2**. To evaluate primer sensitivity, genomic DNA of the novel pathogen was serially diluted tenfold using TE buffer (10 mM Tris-HCl, 1 mM EDTA, pH 8.0) to generate a concentration gradient: 100 ng/μL, 10 ng/μL, 1 ng/μL, 100 pg/μL, 10 pg/μL, and 1 pg/μL. These dilutions were used as templates for PCR amplification under the conditions described above. The detection limit was determined as the lowest DNA concentration that produced a clearly visible and specific band on the agarose gel under these reaction conditions (Lievens et al., 2005).

### 2.6 Screening of biocontrol agents

#### 2.6.1 *In vitro* antagonism assay

The tested biocontrol agents (BCAs), including *Bacillus subtilis*, *Trichoderma harzianum*, and *Streptomyces fungicidicus* strain FYA1, were obtained from the laboratory culture collection. Antagonistic activity was assessed using a dual-culture assay on PDA plates. A 4-mm-diameter mycelial plug from a 3-day-old culture of *P*. *shangluoense* was placed at the center of each plate, and each BCA was streaked linearly 3 cm from the center (Bell et al., 1982; Köhl et al., 2011). For each inoculation, one loopful of mycelia or spores was collected from 5-day-old actively growing cultures to ensure consistent inoculum density across treatments. To investigate the effect of inoculation timing, the BCAs were inoculated at 0 h (simultaneously with the pathogen), 24 h, and 48 h prior to pathogen inoculation. Each treatment combination was replicated six times. Following inoculation of *P*. *shangluoense* at the center of each plate, the plates were incubated at 28 °C under dark conditions for 3 days. After incubation, the inhibition rate of biocontrol agents was calculated based on the radial growth of the pathogen in dual-culture assays (Valetti et al., 2022). In control plates, the radius of the pathogen colony was measured from the center of the plate to the colony margin in the absence of biocontrol agents. In dual-culture plates, the radius of pathogen growth toward the biocontrol agent was measured from the center of the plate to the point of interaction between the pathogen and the antagonist. The inhibition rate was calculated using the following formula:


$$\text{Inhibition rate}\left(\%\right)=\frac{R_{c}-R_{t}}{R_{c}}\times 100\%$$

where *R_c_* represents the radial growth of the pathogen in the control treatment, and *R_t_* represents the radial growth of the pathogen in the presence of the biocontrol agent.

#### 2.6.2 Pot assay for biocontrol efficacy

Based on the *in vitro* antagonism results, *Streptomyces fungicidicus* strain FYA1, which exhibited the strongest inhibitory effect, was selected for the pot assay. For the BCA treatment, solid-state fermentation spore powder of FYA1 (5.6 × 10¹¹ spores/g) was thoroughly mixed with sterilized potting mixture (vermiculite: potting soil = 1:1, v/v) at a rate of 2 g per kg of substrate (w/w). For the control treatment, an equal mass of the sterilized fermentation substrate (primarily composed of composted cattle manure and corn flour) used for FYA1 production was incorporated into the sterilized potting mixture at the same ratio (Huang et al., 2012; Singh et al., 1999). The prepared substrates were dispensed into individual pots (80 g per pot), with 15 pots assigned to each treatment. Seeds of *S*. *miltiorrhiza* were surface-disinfected and germinated on moist sterile filter paper at 25 °C. Upon reaching the cotyledon stage, one healthy seedling was transplanted into each pot. Plants were cultivated in a growth chamber at 25/20 °C (day/night) under a 16 h light/8 h dark photoperiod until they developed four to five true leaves. For pathogen inoculation, each seedling was challenge-inoculated via soil drenching with 20 mL of a *P*. *shangluoense* spore suspension (1 × 10^6^ CFU/mL) (Halo et al., 2019). Following inoculation, plants were maintained under the same environmental conditions for an additional 15–20 days. Disease incidence in each treatment was observed and calculated 15 days post-inoculation.

### 2.7 Data analysis

Statistical analysis was conducted using SPSS 21.0 software (IBM SPSS, Somers, NY, USA). Colony diameters (or radii) of *P. shangluoense* in the temperature and biocontrol assays were first tested for normality using the Shapiro–Wilk test. Homogeneity of variance was assessed using Levene’s test. Data were then analyzed by one-way analysis of variance (ANOVA), followed by the least significant difference (LSD) test for multiple comparisons. Differences in disease incidence between treatments were evaluated using chi-square tests based on the original counts of healthy and diseased plants.

## 3 Results

### 3.1 Isolation and identification of pathogens from diseased *S. miltiorrhiza* roots

Based on colony morphology and ITS sequence analysis, the isolates were identified as belonging to five genera: *Fusarium*, *Alternaria*, *Phytopythium*, *Mucor*, and *Rhizoctonia*. *Fusarium* spp. were the most frequently isolated, with a recovery rate of 45.0%. The dominant species were *F*. *solani* (23.33%) and *F*. *oxysporum* (13.33%) (**Supplementary Table 3**).

**Figure 1:**
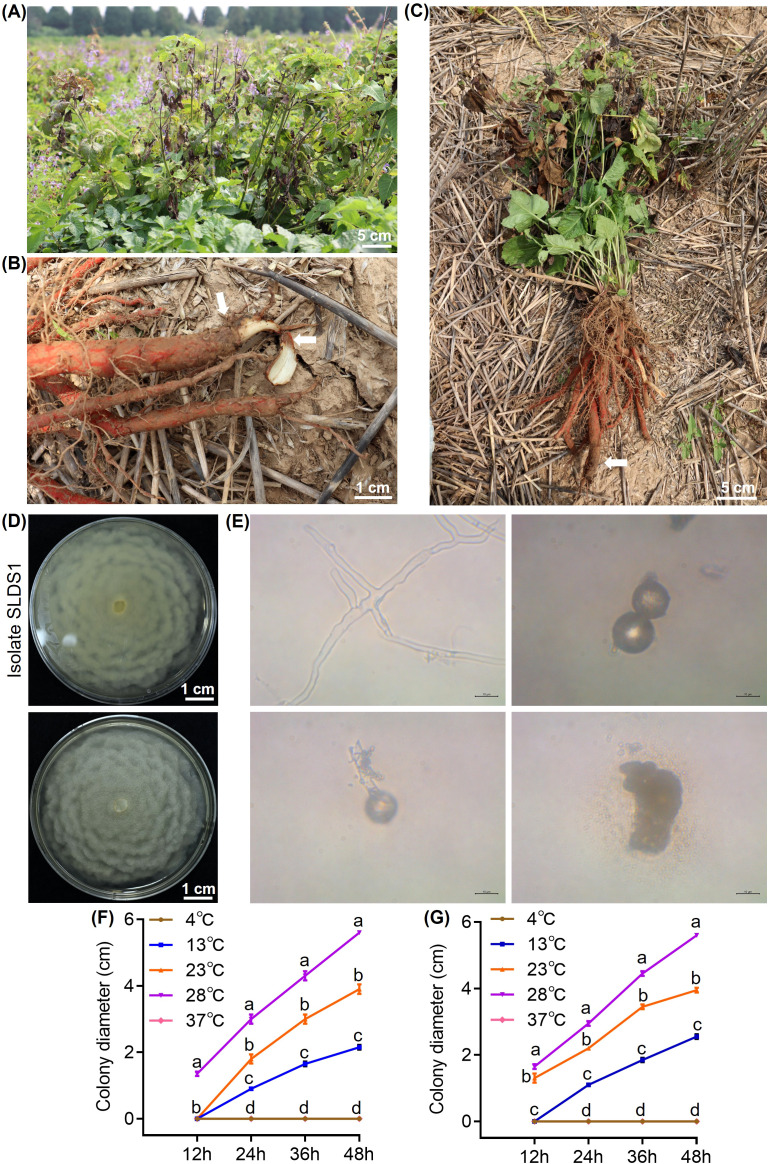
Field symptoms of *Salvia miltiorrhiza* root rot and the morphological and growth characteristics of the novel pathogen. **(A)** Aboveground symptoms showing wilting of an infected plant. **(B)** Belowground symptoms displaying reddish-brown to black rot on the roots. **(C)** Whole-plant view demonstrating both aboveground wilting and belowground root rot. White arrows indicate the diseased parts of the plant. **(D)** Colony morphology (obverse and reverse) of the isolate SLDS1 on V8 medium. **(E)** Microscopic features of the isolate SLDS1: hyphae, sporangia, and spores. Bar = 10 μm. **(F)** Growth curves of isolate SLDS1 on PDA medium incubated at 4 °C, 13 °C, 23 °C, 28 °C, and 37 °C for 5 days. **(G)**. Growth curves of isolate SLDS1 on V8 medium incubated at 4 °C, 13 °C, 23 °C, 28 °C, and 37 °C for 5 days.

Of particular note, a subset of plants from Shangluo exhibited distinct symptoms characterized by systemic wilting of aboveground parts and reddish-brown to black rot of the taproot and stem base (**Figures 1A–C**). The isolate SLDS1 was recovered with high frequency from such symptomatic tissues. On both V8 and PDA media, isolate SLDS1 formed colorless to white, chrysanthemum-like colonies (**Figure 1D**). The hyphae were septate or aseptate. Sporangia were subspherical, with papillae, borne directly on hyphae (**Figure 1E**), and produced spherical zoospores. Temperature assays showed that the isolate SLDS1 grew at temperatures ranging from 13 to 28 °C, with optimal growth at 28 °C, as colony diameters at this temperature were significantly greater than those at other temperatures at each time point (*P* < 0.05). In contrast, no growth was observed at 4 °C or 37 °C after five days of incubation (**Figures 1F, G**). Based on these morphological and cultural characteristics, the isolate SLDS1 was preliminarily identified as belonging to the *genus Phytopythium*. Although root rot caused by *Fusarium*, *Alternaria*, and *Rhizoctonia* on *S*. *miltiorrhiza* has been documented, and our isolation results confirm *Fusarium* as the predominant pathogen (**Supplementary Table 3**), there are no previous reports of *Phytopythium* infecting *S*. *miltiorrhiza* roots. Given that *Phytopythium* species are known to cause severe root rot and damping-off in various crops (Broders et al., 2007; Reeves et al., 2021; Zhang and Yang, 2000), and were consistently associated with distinct symptoms in this study, we focused subsequent investigations on this genus to elucidate its pathogenic role in *S*. *miltiorrhiza*.

### 3.2 Molecular identification of the pathogen

To determine the taxonomic status of the isolate SLDS1, the ITS region of ribosomal DNA was amplified and sequenced. PCR amplification using primers ITS4 and ITS5 yielded a sequence, which was subjected to BLASTn analysis against the NCBI database. The results showed high sequence similarity to various *Phytopythium* species. However, phylogenetic analysis based on the ITS sequence using the ML method revealed that the isolate did not cluster reliably with any known *Phytopythium* species (**Supplementary Figure 1A**).

**Figure 2:**
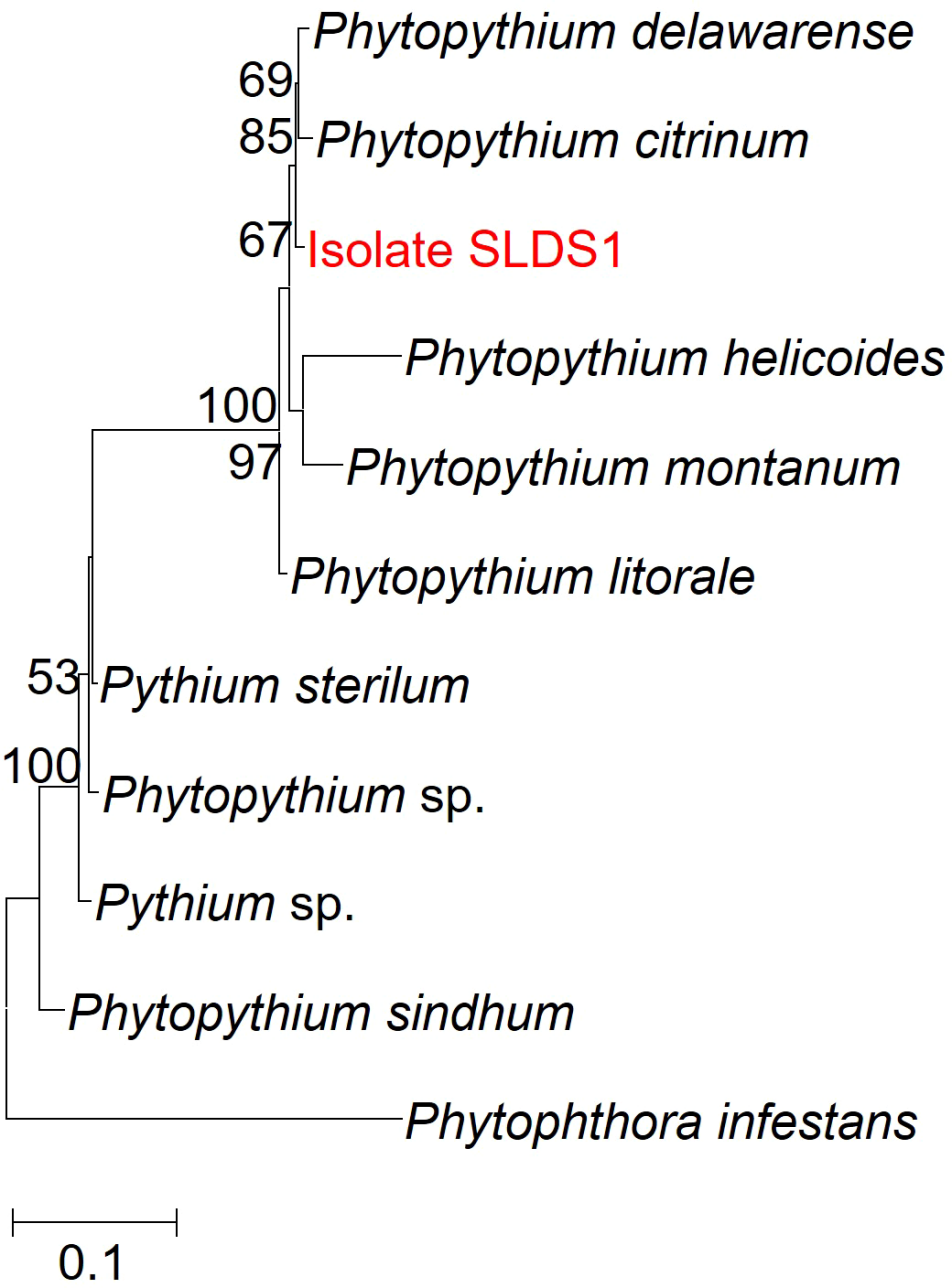
Multigene phylogenetic analyses of the isolate SLDS1 and its closely related species. Maximum Likelihood (ML) trees inferred from the internal transcribed spacer (ITS) region, cytochrome c oxidase subunit I (*cox* I), cytochrome c oxidase subunit II (*cox* II), and *β-tubulin* sequences. The isolate SLDS1 is shown in red. Numbers at nodes represent bootstrap values from 1000 replicates (only values ≥50% are shown). GenBank accession numbers for the sequences used in this analysis are provided in **Supplementary Table 4**.

To further elucidate its phylogenetic position, additional gene regions, including *β-tubulin*, cytochrome c oxidase subunit I (*cox* I), and subunit II (*cox* II), were amplified and sequenced. ML phylogenetic trees were constructed separately based on *tubulin*, *cox* I, and *cox* II sequences. The analyses indicated that the isolate SLDS1 formed a distinct clade separate from all known *Phytopythium* species, suggesting significant genetic divergence (**Supplementary Figures 1B–D**). Subsequently, multi-locus phylogenetic analysis based on concatenated ITS, *cox* I, *cox* II, and *β-tubulin* sequences clearly demonstrated that the unidentified *Phytopythium* isolate SLDS1 forms a distinct and well-supported clade separate from all known species (**Figure 2**; **Supplementary Table 4**). In accordance with its geographical origin (Shangluo, Shaanxi Province, China), it was provisionally designated as *Phytopythium shangluoense* (isolate SLDS1), and has been deposited in the China Center for Type Culture Collection (CCTCC) under accession number CCTCC AF 2026004. The name was registered in MycoBank MB 862769 (https://www.mycobank.org/).

*P. shangluoense* exhibits varying levels of sequence similarity with its closest known relatives, *Phytopythium delawarense* and *Phytopythium citrinum*, across multiple gene loci. In the ITS region, *P. shangluoense* shares 93.92% sequence similarity with *P. delawarense* and 95.09% with *P. citrinum*. For the mitochondrial cytochrome c oxidase subunit I (*cox* I) gene, the novel species shows 89.36% similarity with *P. delawarense* and a notably higher 97.34% similarity with *P. citrinum*. In the *cox* II region, *P. shangluoense* exhibits 95.68% similarity with *P. delawarense* and 94.77% with *P. citrinum*. For the *β-tubulin* gene, the new species demonstrates relatively high similarity with both close relatives: 98.28% with *P. delawarense* and 98.75% with *P. citrinum* (**Supplementary Table 5**). Overall, *P. shangluoense* shows high sequence similarity with *P. citrinum* in the *cox* I and *β-tubulin* genes, while in the ITS and *cox* II regions, it displays comparable similarity levels with both *P. delawarense* and *P. citrinum*.

### 3.3 Pathogenicity analysis

**Figure 3:**
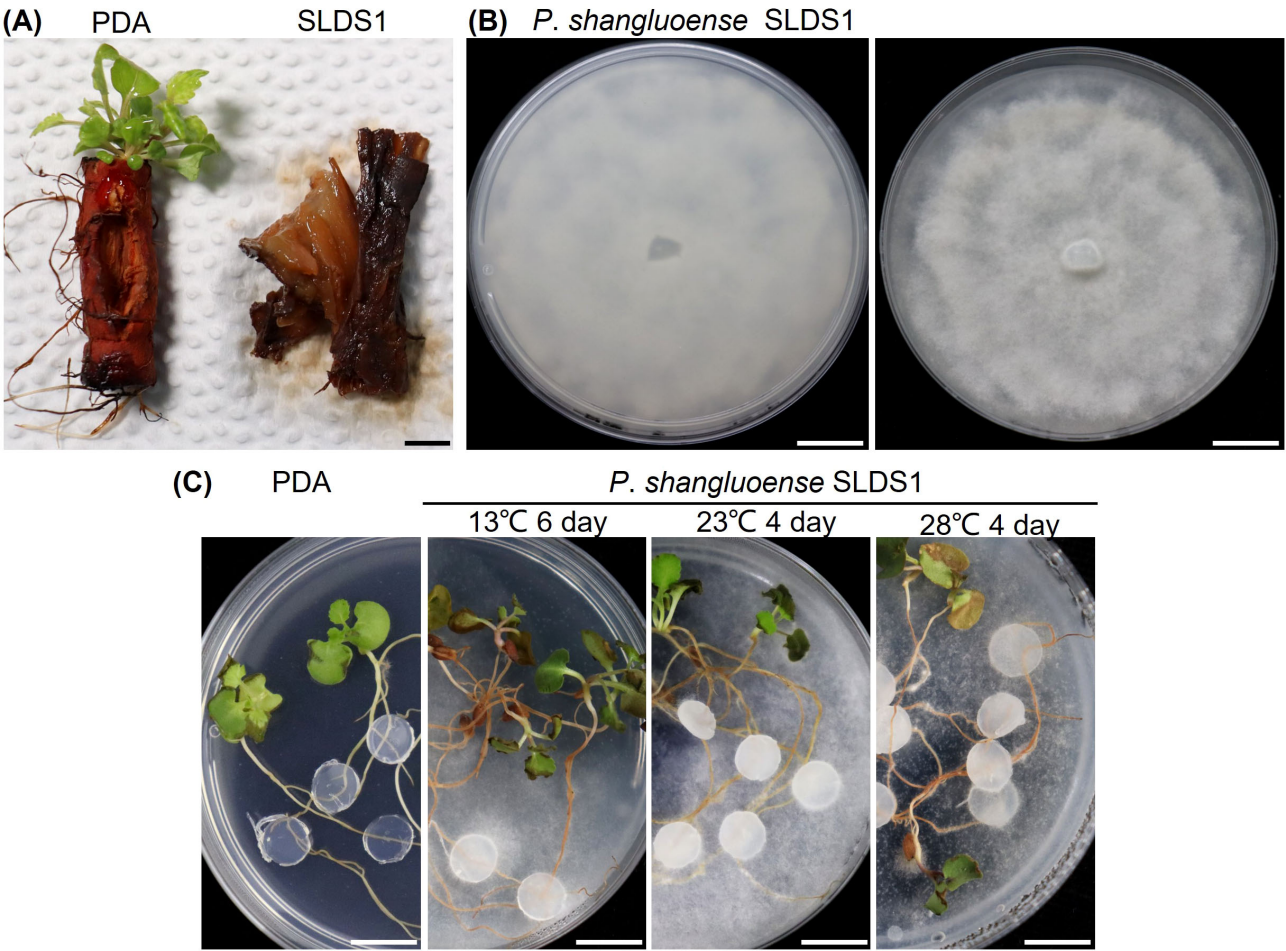
Pathogenicity assay of *Phytopythium shangluoense* SLDS1 on *S. miltiorrhiza*. **(A)** Symptom comparison of detached *S. miltiorrhiza* root segments 20 days after inoculation with *P*. *shangluoense* SLDS1 (right) versus a sterile PDA plug control (left). **(B)** Verification of Koch’s postulates: colony morphology of the pathogen re-isolated from the diseased root segment (panel **(A)**), showing identity with the original inoculum. **(C)** Disease progression in seedlings inoculated with *P*. *shangluoense* SLDS1 at different temperatures: close-up views of root rot on seedlings incubated at 13 °C, 23 °C, and 28 °C. Bar = 1 cm.

To determine the pathogenicity of *P*. *shangluoense* SLDS1 on *S*. *miltiorrhiza*, a detached root inoculation assay was conducted. Following inoculation with mycelial plugs, the root segments were incubated at 25 °C. Disease symptoms, characterized by browning and rot, were first observed at 15 days post-inoculation (dpi). By 20 dpi, all inoculated roots were completely decayed. In contrast, control roots inoculated with sterile PDA plugs remained asymptomatic throughout the observation period (**Figure 3A**). To fulfill Koch’s postulates, the pathogen was re-isolated from typical diseased tissues. The re-isolated strains exhibited colony morphology and microscopic characteristics identical to those of the original *P*. *shangluoense* SLDS1 inoculum, with a 100% recovery rate. This confirmed *P*. *shangluoense* SLDS1 as the causal agent of root rot in *S*. *miltiorrhiza* (**Figure 3B**).

To preliminarily investigate the effect of temperature on disease development, a seedling inoculation experiment was conducted at three temperature regimes: 13 °C, 23 °C, and 28 °C. At 23 °C and 28 °C, all seedlings developed severe root rot within 4 dpi. At 13 °C, disease progression was slightly slower, with 100% incidence reached by 6 dpi. By day 6, root rot was evident in all temperature treatments, accompanied by partial wilting of leaves. Disease severity followed the order: 28 °C > 23 °C > 13 °C (**Figure 3C**).

### 3.4 Development of a specific PCR detection method

**Figure 4:**
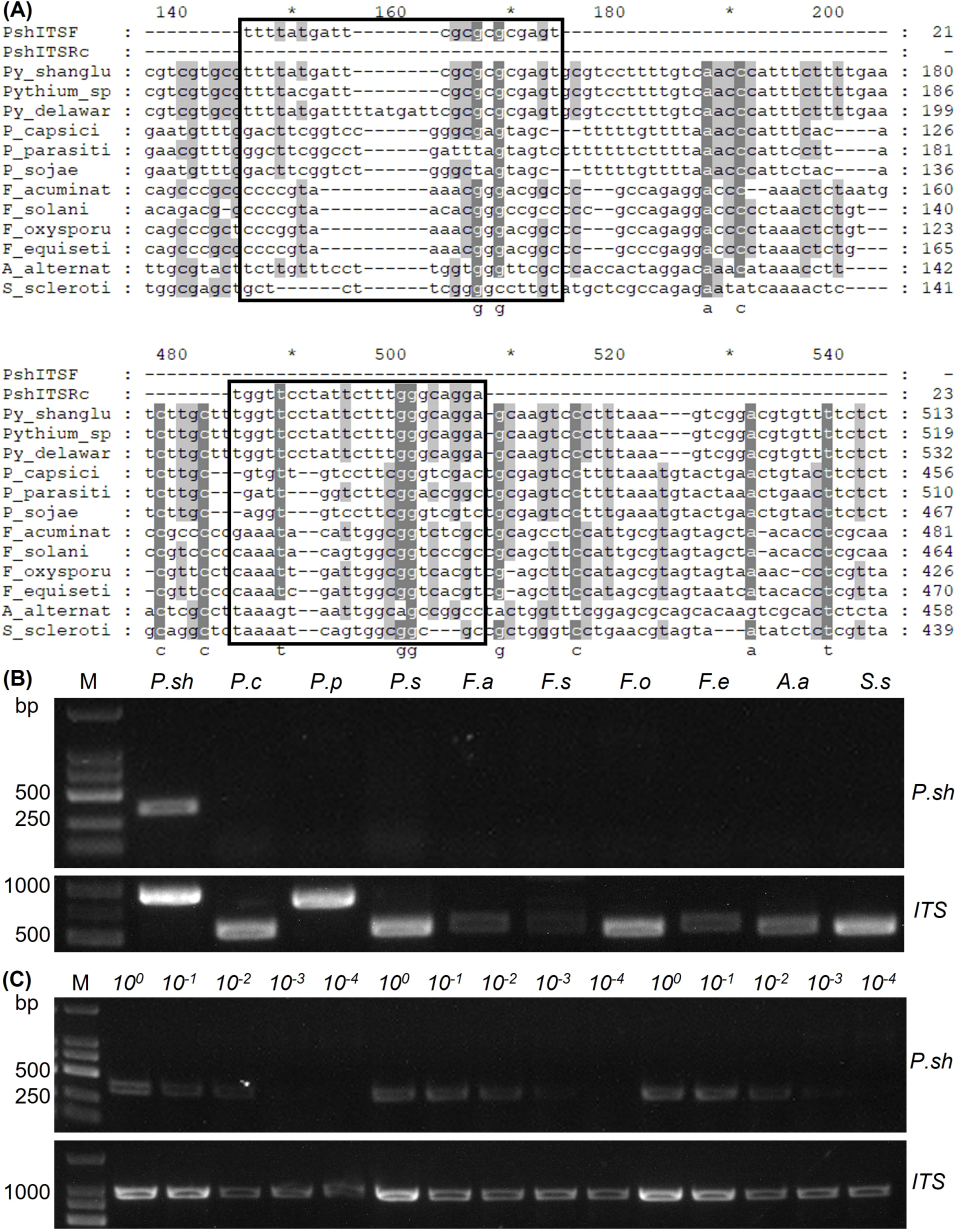
Development of a specific PCR assay for the detection of *Phytopythium shangluoense*. **(A)** Sequence alignment of the ITS region of *P*. *shangluoense* with those of other common pathogens of *S*. *miltiorrhiza*. Arrows indicate the binding sites of the designed specific primers. **(B)** Specificity test: Agarose gel electrophoresis of PCR products amplified using the designed primers from *P*. *shangluoense* (lane 1) and various non-target pathogens (lanes 2-8). *P*. *c*: *Phytophthora capsici, P*. *p*: *Phytophthora parasitica, P*. *s*: *Phytophthora sojae*, *F*. *a*: *Fusarium acuminatum*, *F*. *s*: *Fusarium solani*, *F*. *o*: *Fusarium oxysporum*, *A*. *a*: *Alternaria alternata*, *S*. *s*: *Sclerotinia sclerotiorum*. M: DNA Marker (100~2000 bp) (Sangon Biotech, Shanghai, China; Order NO. B500350), the sizes of relevant bands are indicated on the left in base pairs. **(C)** Sensitivity test: Agarose gel electrophoresis of PCR products using tenfold serially diluted genomic DNA of *P*. *shangluoense* (from 100 ng/μL to 10 pg/μL) as template. The detection limit was 1 ng/μL.

To establish a rapid and sensitive molecular detection method for *P*. *shangluoense*, specific primers were developed based on the ITS region of this pathogen. Through comparative analysis of the ITS sequences of *P*. *shangluoense* with those of common pathogens infecting *S*. *miltiorrhiza*, a unique and conserved region specific to *Phytopythium* spp. was identified. A pair of genus-specific primers was designed, yielding an expected amplicon size of 250 bp (**Figure 4A**; **Supplementary Table 1**). The specificity criterion required that the primers amplify a single band exclusively from *Phytopythium* spp. DNA, with no cross-amplification from other pathogens commonly associated with *S. miltiorrhiza* root diseases. The specificity of the primer set was validated by PCR amplification using genomic DNA from *P*. *shangluoense* and various non-target strains. Analysis of the PCR products by 1.5% agarose gel electrophoresis showed that a single specific band of the expected size was amplified only from *P*. *shangluoense* DNA, with no amplification observed from any non-target strains (**Figure 4B**). Since this is the first report of a *Phytopythium* species causing root rot on *S. miltiorrhiza*, other *Phytopythium* species common in the region were not available for testing. The specificity of the amplified product was further confirmed by direct sequencing and BLAST analysis, which revealed 100% identity with the target sequence.

To evaluate the sensitivity of the assay, genomic DNA of *P*. *shangluoense* was serially diluted tenfold (from 100 ng/μL to 10 pg/μL) and used as template for PCR amplification. The detection limit of the assay was determined to be 1 ng/μL, at which concentration a clear and consistent specific band was observed. At 100 pg/μL, amplification was faint and inconsistent, while no visible amplification occurred at 10 pg/μL (**Figure 4C**). In conclusion, the PCR method developed in this study demonstrated high specificity for *P*. *shangluoense* with a detection sensitivity of 1 ng/μL of genomic DNA.

### 3.5 Screening of Biocontrol Agents and Evaluation of Pot Trial Efficacy

**Figure 5:**
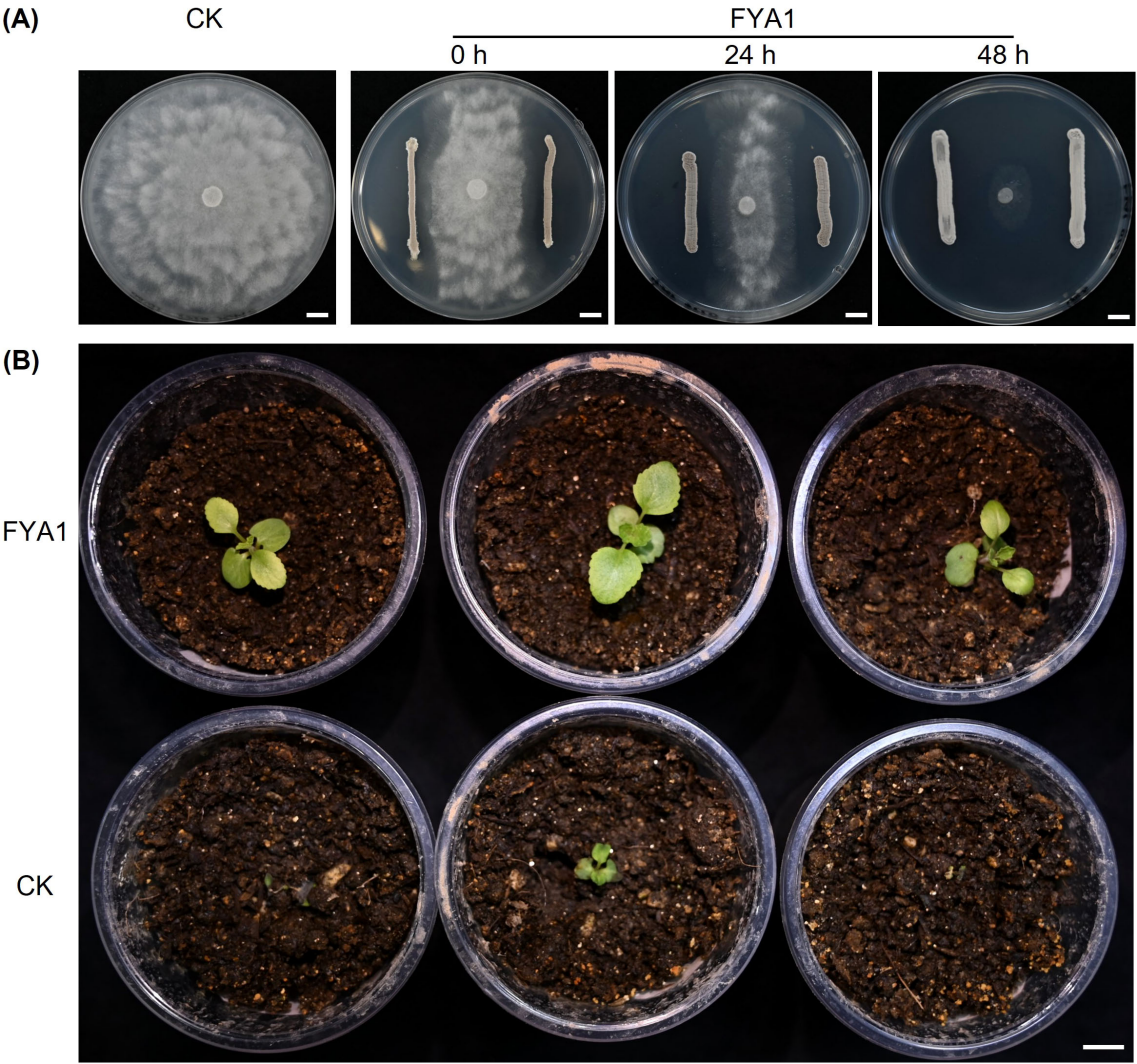
Biocontrol efficacy of *Streptomyces fungicidicus* strain FYA1 against *Phytopythium shangluoense*. **(A)** Inhibition zones against *P*. *shangluoense* produced by the antagonistic strain FYA1, which was streaked on the agar plate 0, 24, and 48 hours prior to pathogen inoculation. Photographs were taken at 96 hours post-inoculation with *P. shangluoense*. **(B)** Disease control effect against *P. shangluoense*-induced root rot in *S*. *miltiorrhiza* by pre-application of strain FYA1 spore powder (2 g/kg substrate, 7 days before pathogen challenge), compared with the substrate-only control (bottom). The pathogen was inoculated at a concentration of 1×10^6^ CFU/mL, 20 mL per plant. Bar = 1 cm.

To screen effective biocontrol agents (BCAs) against the newly identified pathogen *P*. *shangluoense* infecting *S*. *miltiorrhiza*, the *in vitro* antagonistic activities of three BCAs—*Bacillus subtilis*, *Trichoderma harzianum*, and *Streptomyces fungicidicus* strain FYA1—were first assessed using the dual-culture technique (Yang et al., 2024). The results showed that *B*. *subtilis* and *T*. *harzianum* did not produce any visible inhibition zone at any time point, indicating no significant antagonism against *P*. *shangluoense* (**Supplementary Figure 2**). In contrast, strain FYA1 achieved a significant inhibition rate of 42.2% even without pre-inoculation (*P* < 0.05). Importantly, pre-inoculation of FYA1 significantly enhanced its antagonistic effect, with inhibition rates increasing to 63.9% and 88.8% when FYA1 was pre-inoculated 24 h and 48 h prior to pathogen inoculation, respectively (*P* < 0.05; **Figure 5A**; **Supplementary Table 6**), demonstrating a time-dependent enhancement of its antagonistic activity.

Based on these *in vitro* results, FYA1, which showed the strongest antagonism, was selected for a pot assay. Disease assessment indicated that, at 15 days post pathogen inoculation, disease incidence in the control reached 100%, with plants exhibiting severe rot symptoms leading to whole-plant decay. In contrast, disease incidence in the FYA1-treated group was significantly reduced to 20.0% (χ² = 16.81, *df* = 1, *P* < 0.001), and affected plants displayed only mild symptoms (**Figure 5B**; **Supplementary Table 7**). The biocontrol efficacy of FYA1 was calculated to be 80.0%. These results demonstrate that *Streptomyces fungicidicus* strain FYA1 exhibits significant antagonistic activity against *P*. *shangluoense* and effectively controls root rot caused by this pathogen in *S*. *miltiorrhiza* under pot conditions.

## 4 Discussion

*S*. *miltiorrhiza* root rot represents a major constraint to the sustainable development of the *S*. *miltiorrhiza* industry, and its pathogen composition varies among production regions (Sa et al., 2022; Yang et al., 2021a). In the present study, a novel *Phytopythium* species, *Phytopythium shangluoense*, was identified from the Shangluo production area in Shaanxi Province and confirmed as an important causal agent of local *S*. *miltiorrhiza* root rot, thereby expanding the known pathogen spectrum associated with this disease. In contrast to major production areas such as Shandong and Henan Provinces, where *Fusarium* spp. are reported as the predominant pathogens (Li et al., 2022a; Sa et al., 2022; Wang et al., 2018; Yang et al., 2021b), root rot in the Shangluo region was characterized by a complex infection involving both *Fusarium* spp. and *Phytopythium* spp. Our finding demonstrates that the pathogen composition of *S*. *miltiorrhiza* root rot is strongly region-dependent, which may be closely associated with differences in topography, precipitation patterns, and soil moisture conditions among regions (Delavaux et al., 2021). The mountainous terrain of the Shangluo area, together with relatively concentrated rainfall, results in persistently high soil moisture levels that are more favorable for the survival and infection of *Phytopythium* species (Amaresan and Kumar, 2025; Zhao et al., 2007).

From a pathogen biology perspective, accurate identification and characterization of biological traits are essential for understanding disease development and pathogenic behavior. In this study, morphological characteristics combined with multigene phylogenetic analyses based on ITS, *β-tubulin*, *cox* I, and *cox* II sequences demonstrated that the isolates did not correspond to any previously described *Phytopythium* species. The pathogen was therefore designated as a novel species, *P*. *shangluoense*, named based on its place of isolation (**Figure 2**). Its cultural characteristics, including colony morphology and sporangial features, as well as its temperature growth range (13 to 28 °C, with an optimum at 28 °C), were consistent with members of the *Globosum* subclade of *Phytopythium* (Tai, 1981).

Pathogenicity assays further confirmed that *P*. *shangluoense* SLDS1 was capable of infecting detached roots and seedlings of *S*. *miltiorrhiza*, exhibiting pathogenicity across a temperature range of 13 to 28 °C with clear temperature dependence, and causing the most severe disease symptoms at 28 °C (**Figure 3**). This temperature response is consistent with previous reports indicating that *Phytopythium* species generally maintain pathogenic activity at relatively low temperatures, whereas many *Fusarium* spp. tend to be more aggressive under relatively higher temperatures (You and Tojo, 2020). Based on these observations, it is reasonable to infer that the broad temperature adaptability of *P*. *shangluoense* may facilitate complementary seasonal infection dynamics with thermophilic *Fusarium* pathogens, thereby extending the overall infection period. This interaction may partly explain the longer disease occurrence period of *S*. *miltiorrhiza* root rot observed in the Shangluo region compared with other major production areas such as Shandong and Sichuan Provinces (Liu et al., 2022a; Luo et al., 2021). These findings highlight the importance of considering the spatiotemporal dynamics of pathogen assemblages when developing effective disease management strategies.

Given the potential threat posed by *P*. *shangluoense* as an emerging pathogen, the development of rapid and species-specific detection methods is essential for early disease warning and targeted management. In this study, species-specific primers designed based on the ITS region of *P*. *shangluoense* exhibited high specificity in PCR assays, amplifying only the target pathogen, and showed good sensitivity with a detection limit of 1 ng/μL (**Figure 4**). These results further indicate that the ITS region harbors localized, species-specific nucleotide polymorphisms in *P*. *shangluoense*. This detection tool can be applied for monitoring *P*. *shangluoense* in field soils, planting materials, and diseased tissues, thereby providing guidance for disease management decisions and the optimization of control timing.

Previous biocontrol studies on *S*. *miltiorrhiza* root rot have largely focused on antagonists targeting *Fusarium* spp., particularly *Bacillus* spp. and *Trichoderma* spp (Ming et al., 2013; Sa et al., 2022; Wang et al., 2023; Yan et al., 2011). However, plate confrontation assays in the present study showed that *B*. *subtilis* and *T*. *harzianum* exhibited no significant antagonistic activity against *P*. *shangluoense* (**Supplementary Figure 2**), whereas *Streptomyces fungicidicus* strain FYA1 exhibited strong, time-dependent inhibitory effects *in vitro*. Subsequent pot assays further confirmed its effective disease control performance (**Figure 5**; **Supplementary Table 6**). These results highlight the necessity of pathogen-specific screening when developing biocontrol strategies. Notably, strain FYA1 has also been reported to exhibit strong antagonistic activity against other pathogens, including *Sclerotium rolfsii* and *Fusarium* spp (Li et al., 2022b), further underscoring its potential for managing *S*. *miltiorrhiza* root rot caused by multiple pathogens.

Based on these findings, several implications for the management of *S*. *miltiorrhiza* root rot, particularly cases involving *Phytopythium* infection, can be proposed. First, disease monitoring programs should incorporate genus-specific detection of *Phytopythium* pathogens to clarify their distribution and epidemiological dynamics. Second, biocontrol practices should prioritize the selection or combination of antagonists targeting locally dominant pathogens; in this context, the field performance of strain FYA1 warrants further evaluation. Third, agricultural and ecological management strategies should be strengthened, especially through improved drainage practices to reduce soil moisture, thereby creating conditions less favorable for the proliferation and spread of soilborne pathogens such as *Phytopythium* spp.

Nevertheless, several limitations of the present study should be acknowledged. The infection cycle, key pathogenicity determinants, and host–pathogen interaction mechanisms of *P*. *shangluoense* remain to be elucidated. In addition, the interactions between *P*. *shangluoense* and other root rot pathogens, including *Fusarium* spp., under field conditions—whether synergistic, competitive, or antagonistic—require further investigation, as these interactions are critical for understanding the nature of disease complexes.

## Data Availability

The original contributions presented in the study are included in the article/**Supplementary Material**. Further inquiries can be directed to the corresponding author.
