# Supplementary material for: A novel Phytopythium species causing root rot of Salvia miltiorrhiza in China: pathogenicity, molecular identification, and biocontrol potential
Source: Front Plant Sci. 2026 Apr 24;17:1803698. doi: 10.3389/fpls.2026.1803698 (PMC13153048; doi:10.3389/fpls.2026.1803698)
Supplement: Supplementary file 1 [file DataSheet1.docx]

Supplementary Material

# Supplementary Figures and Tables

## Supplementary Figures


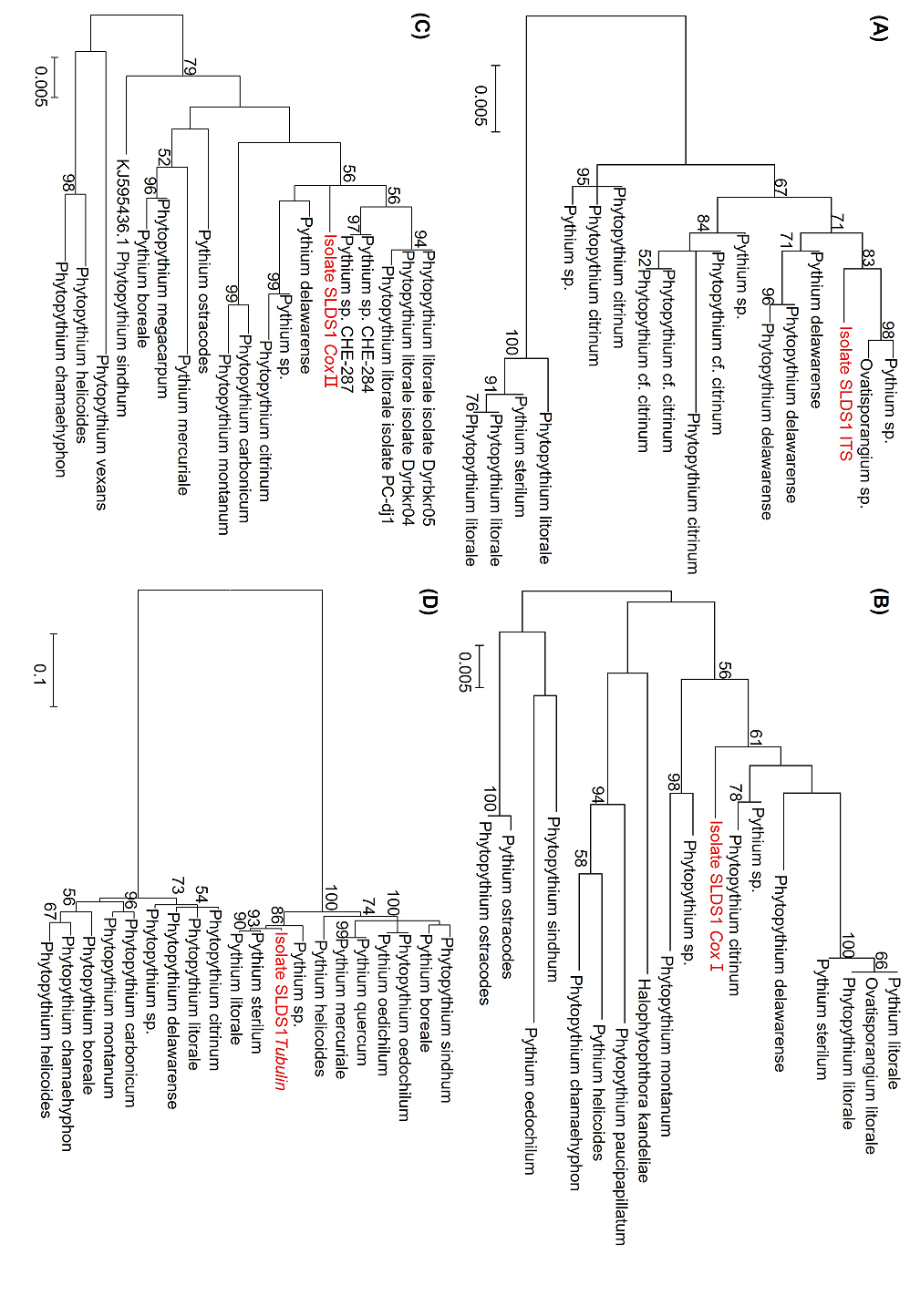


**Supplementary Figure S1. Phylogenetic analysis based on multilocus gene sequences*.***Maximum Likelihood phylogenetic trees based on internal transcribed spacer (A), cytochrome c oxidase subunit I (B), cytochrome c oxidase subunit Ⅱ (C), and *β-tubulin* (D) gene sequences. The isolate SLDS1 is shown in red. Numbers at nodes represent bootstrap values from 1000 replicates (only values ≥50% are shown).


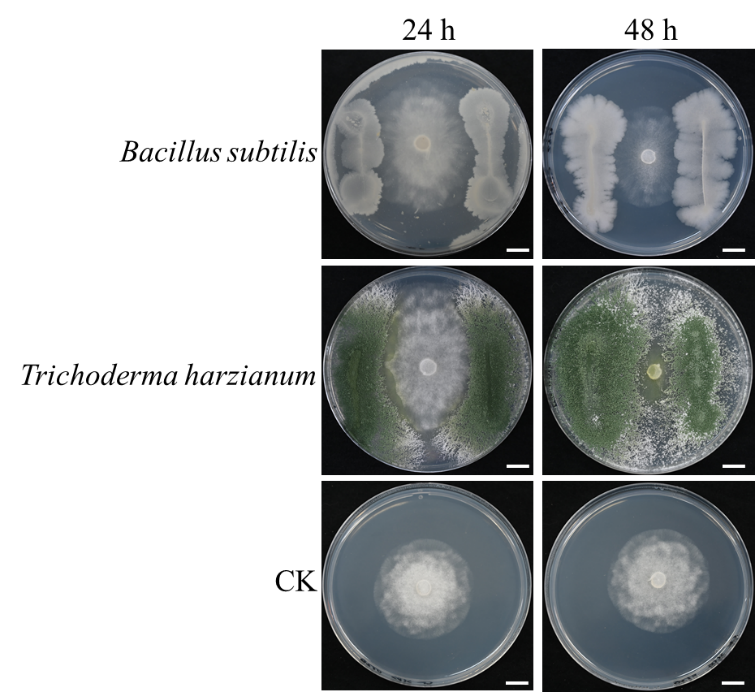


**Supplementary Figure S2. Inhibitory effects of *Bacillus subtilis* and *Trichoderma harzianum* against *Phytopythium shangluoense* SLDS1*.***The figure shows the antagonistic activity of two common biocontrol agents—*B. subtilis* and *T. harzianum*—against the pathogen *P. shangluoense* SLDS1 when streaked 24 and 48 hours prior to pathogen inoculation. Photographs were taken at 48 hours post-inoculation with *P. shangluoense*. No distinct inhibition zone was observed for either biocontrol agent at the tested time points. Bar = 1 cm.

## Supplementary Tables

**Supplementary Table S1.** Primers used in this study.

| Target DNA | Primer name | Primer sequence (5ʹ→3ʹ) | Reference |
| --- | --- | --- | --- |
| *Psh*ITS | *Psh*ITS*-F* | TTTTATGATTCGCGCGCGAGT | Designed |
|  | *Psh*ITS*-R* | TCCTGCCCAAAGAATAGGAACCA |  |
| *Tubulin* | *Tubulin-F* | TGYCCDTCGCCNAAGGTGTCGGA | Villa et al., 2006 |
|  | *Tubulin-R* | GGAACATBGCCGTGAACTGCT |  |
| *Cox* I | *Cox* I*-F* | ATAAWYTCYYATCATGATWTAATG | Chen et al., 2016 |
|  | *Cox* I*-R* | TTTGATTTAAACGACCWGGACAA |  |
| *Cox* II | *Cox* II*-F* | GGC AAA TGG GTT TTC AAG ATC C | Chen et al., 2016 |
|  | *Cox* II*-RC4* | TGA TTW AYN CCA CAA ATT TCR CTA CAT TG |  |
| ITS | ITS4 | TCCTCCGCTTATTGATATGC | Sahar, 2016 |
|  | ITS5 | GGAAGTAAAAGTCGTAACAAGG |  |

**Supplementary Table S2.** The GenBank accession numbers for the sequences used in the specificity validation.

| Species | GenBank accession No. (ITS) | Classification |
| --- | --- | --- |
| *Phytopythium shangluoense* | PZ052490 | Oomycota |
| *Pythium* sp. | AB468792.1 | Oomycota |
| *Phytopythium delawarense* | AB725875.1 | Oomycota |
| *Phytophthora capsici* | AB217670.1 | Oomycota |
| *Phytophthora parasitica* | AY713471.1 | Oomycota |
| *Phytophthora sojae* | AB217685.1 | Oomycota |
| *Fusarium acuminatum* | PQ483290.1 | Fungi |
| *Fusarium solani* | PP059531.1 | Fungi |
| *Fusarium oxysporum* | OR734798.1 | Fungi |
| *Fusarium* *equiseti* | MZ496590.1 | Fungi |
| *Alternaria alternata* | OL958426.1 | Fungi |
| *Sclerotinia sclerotiorum* | JQ480621.1 | Fungi |

**Supplementary Table S3.** Composition and isolation frequency of pathogens obtained from root rot samples of *Salvia miltiorrhiza.*

| Pathogen types | *Fusarium* | *Alternaria* | *Phytopythium* | *Mucor* | *Rhizoctonia* |
| --- | --- | --- | --- | --- | --- |
| Number of isolates (100%) | 81(45%) | 30(16.67%) | 31(17.22%) | 24(13.33%) | 14(7.78%) |

**Supplementary Table S4.** Species names and GenBank accession numbers for sequences used in this study.

| Species | GenBank Accession Number | | | |
| --- | --- | --- | --- | --- |
|  | ITS | *Cox* I | *Cox* II | *Tubulin* |
| *Phytopythium* sp. | PP812163.1 | OR900699.1 | PV239525.1 | PV269611.1 |
| *Pythium* sp. | AB468792.1 | EF406100.1 | EU199108.1 | EU199092.1 |
| *Phytopythium delawarense* | AB725875.1 | KF853240.1 | AB690672.1 | AB948181.1 |
| *Phytopythium litorale* | OR531132.1 | OP903812.1 | MW208003.1 | PV638057.1 |
| *Phytopythium citrinum* | HQ643377.1 | OP903811.1 | AB690679.1 | AB948180.1 |
| *Phytopythium helicoides* | PQ200672.1 | MT222457.1 | MN952211.1 | AB948185.1 |
| *Phytopythium montanum* | PX593151.1 | HQ708438.1 | KJ595410.1 | AB948184.1 |
| *Phytopythium sindhum* | MG799211.1 | HM244822.1 | KJ595436.1 | KJ595559.1 |
| *Pythium sterilum* | HQ398249.1 | EF406093.1 | EF406091.1 | EF421186.1 |
| *Phytophthora infestans* | OR253485.1 | MN458152.1 | DQ365743.1 | AY564037.1 |
| *Phytopythium shangluoense* | PZ052490 | PZ052539 | PZ055799 | PZ055800 |

**Supplementary Table S5.** Multi-gene sequence similarity comparison between *Phytopythium shangluoense* and its closely related species *P. delawarense* and *P. citrinum*.

| Gene | *P*. *shangluoense* vs. *P*. *delawarense* | *P*. *shangluoense* vs. *P*. *citrinum* |
| --- | --- | --- |
| ITS | 93.92% | 95.09% |
| *Cox I* | 89.36% | 97.34% |
| *Cox II* | 95.68% | 94.77% |
| *Tubulin* | 98.28% | 98.75% |

**Supplementary Table S6.** *In vitro* antagonistic effects of *Streptomyces fungicidicus* strain FYA1 against *Phytopythium shangluoense* SLDS1.

| Treatment | Radius of the pathogen (mm)^1^ | Inhibition rate (%) |
| --- | --- | --- |
| Control | 42.9±1.04a | — |
| FYA1 (0h) | 24.8±0.19c | 42.2 |
| FYA1 (24h) | 15.5±0.11b | 63.9 |
| FYA1 (48 h) | 4.8±0.23d | 88.8 |
| ^1^n = 6 biological replicates. Values are means ± standard deviation. The Shapiro–Wilk test and Levene’s test indicated that the data met the assumptions of normality and homogeneity of variance, respectively (*P* > 0.05). Different letters indicate significant differences among treatments (P < 0.05). | | |

**Supplementary Table S7.** Disease incidence in pot trials with *Streptomyces fungicidicus* strain FYA1 against *Phytopythium shangluoense*-induced root rot in *Salvia miltiorrhiza*.

| Treatment | Incidence rate^1^ |
| --- | --- |
| CK | 100% |
| FYA1 | 20.00% |
| ^1^Differences in disease incidence between the control and treatment groups were evaluated using a Chi-square test (χ² = 16.81, *df* = 1, *P* < 0.001). | |
